# A Proteomics-Based Assessment of Inflammation Signatures in Endotoxemia

**DOI:** 10.1074/mcp.RA120.002305

**Published:** 2021-02-24

**Authors:** Sean A. Burnap, Ursula Mayr, Manu Shankar-Hari, Friederike Cuello, Mark R. Thomas, Ajay M. Shah, Ian Sabroe, Robert F. Storey, Manuel Mayr

**Affiliations:** 1 King's College London British Heart Foundation Centre, School of Cardiovascular Medicine and Sciences, London, United Kingdom; 2 School of Immunology and Microbial Sciences, King's College London and Guy’s and St Thomas’ NHS Foundation Trust, London, United Kingdom; 3 Department of Experimental Pharmacology and Toxicology, Cardiovascular Research Centre, University Medical Centre Hamburg-Eppendorf, Hamburg, Germany; 4 DZHK (German Center for Cardiovascular Research), partner site Hamburg/Kiel/Lübeck, University Medical Center Hamburg-Eppendorf, Hamburg, Germany; 5 Institute of Cardiovascular Sciences, University of Birmingham, Birmingham, United Kingdom; 6 Sheffield Pulmonary Vascular Disease Unit, Royal Hallamshire Hospital, Sheffield, United Kingdom; 7 Cardiovascular Research Unit, Department of Infection Immunity and Cardiovascular Disease, University of Sheffield, Sheffield, United Kingdom

**Keywords:** Proteomics, pentraxin-3, inflammation, endotoxin neutrophils, sepsis, biomarker, ApoA2, apolipoprotein-A2, CRP, C-reactive protein, DDA, data-dependent acquisition, DIA, data-independent acquisition, ECM, extracellular matrix, FA, formic acid, FDR, false discovery rate, HDL, high-density lipoprotein, ICAM1, intercellular adhesion molecule 1, iRT, indexed retention time, KO, knock-out, LBP, lipopolysaccharide-binding protein, LPS, lipopolysaccharide, MPO, myeloperoxidase, Nox2, NADPH oxidase 2, PBST, PBS containing 0.1% Tween-20, PTX3, pentraxin-3, RT, retention time, SAA1, serum amyloid A-1, SAA2, serum amyloid A-2, TMT, tandem mass tag, VCAM-1, vascular cell adhesion molecule-1

## Abstract

We have previously shown that multimers of plasma pentraxin-3 (PTX3) were predictive of survival in patients with sepsis. To characterize the release kinetics and cellular source of plasma protein changes in sepsis, serial samples were obtained from healthy volunteers (*n* = 10; three time points) injected with low-dose endotoxin (lipopolysaccharide [LPS]) and analyzed using data-independent acquisition MS. The human plasma proteome response was compared with an LPS-induced endotoxemia model in mice. Proteomic analysis of human plasma revealed a rapid neutrophil degranulation signature, followed by a rise in acute phase proteins. Changes in circulating PTX3 correlated with increases in neutrophil-derived proteins following LPS injection. Time course analysis of the plasma proteome in mice showed a time-dependent increase in multimeric PTX3, alongside increases in neutrophil-derived myeloperoxidase (MPO) upon LPS treatment. The mechanisms of oxidation-induced multimerization of PTX3 were explored in two genetic mouse models: MPO global knock-out (KO) mice and LysM Cre Nox2 KO mice, in which NADPH oxidase 2 (Nox2) is only deficient in myeloid cells. Nox2 is the enzyme responsible for the oxidative burst in neutrophils. Increases in plasma multimeric PTX3 were not significantly different between wildtype and MPO or LysM Cre Nox2 KO mice. Thus, PTX3 may already be stored and released in a multimeric form. Through *in vivo* neutrophil depletion and multiplexed vascular proteomics, PTX3 multimer deposition within the aorta was confirmed to be neutrophil dependent. Proteomic analysis of aortas from LPS-injected mice returned PTX3 as the most upregulated protein, where multimeric PTX3 was deposited as early as 2 h post-LPS along with other neutrophil-derived proteins. In conclusion, the rise in multimeric PTX3 upon LPS injection correlates with neutrophil-related protein changes in plasma and aortas. MPO and myeloid Nox2 are not required for the multimerization of PTX3; instead, neutrophil extravasation is responsible for the LPS-induced deposition of multimeric PTX3 in the aorta.

## Highlights

• Multimer formation of pentraxin-3 (PTX3) has been linked to outcome in sepsis.
• PTX3 plasma levels have been correlated to increases in neutrophil-derived proteins.
• Neutrophil-derived myeloperoxidase and NADPH oxidase 2 are not responsible for PTX3 oxidation.
• PTX3 multimer deposition within the aorta is neutrophil dependent.

## Graphical Abstract

**Figure:**
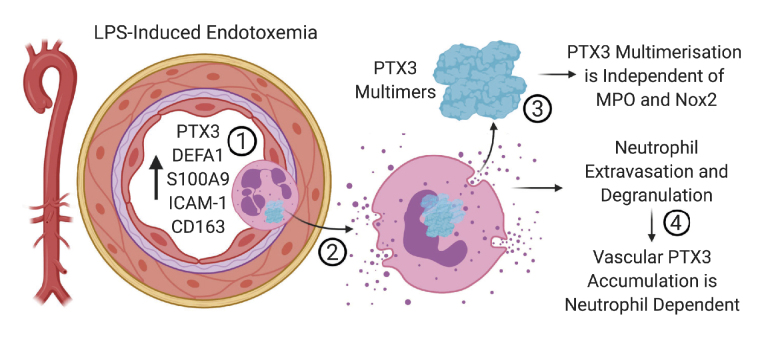

## In Brief

Circulating proteome alterations, in both humans and mice, in response to endotoxin were mapped utilizing proteomic approaches. Multimeric pentraxin-3 (PTX3) plasma and aortic levels mimicked changes observed in proteins of known neutrophil origin in response to endotoxin. Genetic mouse models ruled out myeloperoxidase and NADPH oxidase 2 as drivers of PTX3 oxidation; however, neutrophil extravasation promotes PTX3 deposition within the vasculature. The results reveal a link between the systemic inflammatory response and the neutrophil-dependent vascular deposition of multimeric PTX3.

The human immune response to infection and injury has high interindividual variation at a cellular and transcriptome level (1). There is well-known discordance between mRNA expression and protein levels (2). Therefore, identifying proteomic features will further our understanding of the immune response (3, 4). Information, however, on how defined inflammatory signals, such as endotoxin (LPS, lipopolysaccharide), alter the human plasma proteome is currently lacking.

Moreover, many inflammatory proteins form protein complexes. For example, long pentraxin-3 (PTX3) is a critical mediator of the innate immune system, capable of binding microbial moieties acting as a pattern-recognition receptor, leading to the opsonization of bound bacterial and fungal components, subsequently promoting their clearance by immune cells (5, 6, 7). PTX3 forms homomultimeric structures through interdisulfide bond formation that determines its ability to bind members of the complement system (8, 9). While total PTX3 levels in plasma are associated with outcomes after acute coronary syndrome, closely correlating with known markers of myocardial damage (10), we have previously shown that multimeric forms of PTX3 were superior to the measurement of total PTX3 levels alone in predicting mortality in patients with sepsis (11). However, key regulators of PTX3 multimer formation remain unknown.

In the present study, we mapped the human immune response to low-dose endotoxemia over time, through the use of data-independent acquisition (DIA) techniques. Furthermore, using murine models of endotoxemia and multiplexed proteomics, we assessed the contribution of key oxidant-generating enzymes in the regulation of PTX3 multimerization and reveal that vascular deposition of PTX3 in response to sepsis is neutrophil dependent.

## Experimental Procedures

### Human Study Population

This prospective, randomized, and open-label study was approved by the Sheffield Research Ethics Committee (UK) and the Medicines and Healthcare Products Regulatory Agency (UK) and was conducted in accordance with Good Clinical Practice guidelines. Subjects provided written informed consent. Inclusion criteria were age older than 18 years with no significant medical issues, no regular use of medication, and willingness to abstain from consuming caffeine (an adenosine receptor antagonist). Exclusion criteria included any clinically significant abnormality detected on screening (medical history, physical examination, ECG, and routine blood tests), recent blood donation or vaccination, a history of alcohol or drug abuse, or a contraindication to study medication. The study was registered at http://www.clinicaltrials.gov (unique identifier: NCT01846559) (12).

### Human Experimental Protocol

The control arm (*n* = 10) of the aforementioned study was used; all study participants were male (12). One venous cannula was inserted into an antecubital vein in each arm. One cannula was used for blood sampling, and the other cannula was used for administration of LPS and intravenous fluid (250 ml 0.9% saline over 30 min prior to LPS administration, then 500 ml 0.9% saline over 4 h after LPS administration). About 2 ng/kg *Escherichia coli* O:113 LPS (Clinical Center Reference Endotoxin, National Institutes of Health) was administered over 1 min at *t* = 0 h. Venous blood samples were collected at baseline (prior to any randomized medication), prior to LPS administration, and at the following time points after LPS administration: 5, 15, and 30 min and 1, 1.5, 2, 4, 6, and 24 h. Baseline, 6 h, and 24 h time points were used for proteomics in this study. Blood samples for isolation of plasma were collected into tubes containing trisodium citrate dihydrate (3.13% w/v), centrifuged immediately at 1500*g* for 10 min, and the supernatant stored at −80 °C. Plasma aliquots used for proteomic analysis had less than three freeze–thaw cycles. All laboratory measurements were performed by staff blinded to treatment allocation.

### Animal Models

All experiments were performed in accordance with UK Home Office regulations, and the investigation conformed with the Guide for the Care and Use of Laboratory Animals published by the US National Institutes of Health (National Institutes of Health publication no.: 85-23, revised 1996). C57BL/6J, global myeloperoxidase (MPO) knock-out (KO) and LysM Cre NADPH oxidase 2 (Nox2) KO mice were injected i.p. with 9 mg/kg LPS (serotype 0.11:B4, Sigma-Aldrich). Control animals received intraperitoneal injections with an equivalent volume of saline. Mice were sacrificed 1, 2, 3, and 4 h after injection (*n* = 3 or 4 biological replicates per time point). To achieve depletion of neutrophils, mice were injected i.p. with 200 μg antimouse Ly-6G antibody (*n* = 4) (Clone 1A8; BioLegend); control mice were injected with 200 μg rat IgG2a isotype control antibodies (*n* = 5) (BioLegend). About 20 h after antibody injection, mice were injected i.p. with 9 mg/kg LPS and sacrificed 4 h after injection. Blood samples for isolation of plasma were collected into tubes containing trisodium citrate dihydrate (3.13% w/v), an aliquot was taken for immediate RNA isolation, and the remainder was centrifuged at 1500*g* for 10 min and the supernatant stored at −80 °C. Tissue samples, 10 to 20 mg, were washed in ice-cold PBS to remove blood contamination before homogenization in the presence of tissue lysis buffer (25 mM Tris–HCl, 110 mM NaCl, 2 mM EGTA, 5 mM EDTA, 1% Triton, and 0.1% SDS) containing protease inhibitor cocktail (Roche) at pH 7.4. Cellular debris was then pelleted by centrifugation, 16,000*g*, 15 min at 4 °C. Protein concentration was measured using a BCA protein assay kit (Thermo Fisher Scientific).

### Plasma Depletion and In-solution Protein Digestion

Human plasma was depleted using High-Select top14 abundant protein depletion columns (Thermo Fisher Scientific), whereas mouse plasma was depleted of the top7 abundant proteins using Seppro spin columns (Merck), following manufacturer's instructions. Protein samples were denatured by the addition of a final concentration of 6 M urea and 2 M thiourea and reduced by the addition of a final concentration of 10 mM DTT followed by incubation at 37 °C for 1 h, 240 rpm. The samples were then cooled down to room temperature before being alkylated by the addition of a final concentration of 50 mM iodoacetamide followed by incubation in the dark for 30 min. Prechilled (−20 °C) acetone (10× volume) was used to precipitate the samples overnight at −20 °C. Samples were centrifuged at 14,000*g* for 40 min at 4 °C, and the supernatant subsequently discarded. Protein pellets were dried using a SpeedVac system (Thermo Scientific; Savant SPD131DDA), resuspended in 0.1 M triethylamine bicarbonate buffer, pH 8.0, containing 0.02% ProteaseMax surfactant and MS grade trypsin/Lys-C (Promega Cooperation) (1:25 enzyme:protein), and digested overnight at 37 °C, 240 rpm. An aliquot of peptide digest was taken for tandem mass tag (TMT) labeling, while the digestion was stopped for label-free DIA analyses by acidification with TFA. Peptides were then purified robotically, using C18 cartridges (Bravo AssayMAP; Agilent Technologies).

### Experimental Design and Statistical Rationale

The mouse LPS time course plasma and tissue TMT analyses were divided across two TMT-10plex sets in each experiment, containing either three or four biological replicates per time point, and replicates per time point were split equally across TMT groups to avoid technical bias. A common pooled calibrator sample was also included for all TMT experiments to enable crossgroup comparisons. The mouse neutrophil depletion experiment contained either five or four biological replicates per group and was analyzed using a TMT-10plex, with the inclusion of a pooled sample. Human DIA analyses with 30 samples in total from ten individuals at three time points were conducted as follows: samples at each time point per individual were injected for MS analysis sequentially, with a wash gradient in between each individual, enabling an accurate determination of protein changes induced by LPS injection over the time course per individual. To determine analytical reliability, variation in protein abundances across standard HeLa digest (Pierce; Thermo Scientific) injections were determined and compared with the variation in protein abundances across the cohorts analyzed, on both the Q-Exactive HF and Orbitrap Fusion Lumos Tribrid. Technical variation on both mass spectrometers was below 15% (supplemental Fig. S1). To determine reproducibility and validity of DIA findings, nondepleted plasma samples from the human cohort were analyzed by label-free quantification, and significantly changing proteins over the LPS time course were compared (supplemental Fig. S2).

### TMT Peptide Labeling

Plasma and tissue peptide samples were TMT-10plex labeled according to the manufacturer's instructions. Samples and pool were then grouped in equal amounts, ensuring randomization, and dried using a SpeedVac system (Thermo Fisher Scientific; Savant SPD131DDA). Grouped TMT samples were then reconstituted with 0.1% TFA in H_2_O, for peptide fractionation using high pH reversed-phase spin columns, following the manufacturer's instructions (Thermo Fisher Scientific).

### LC–MS/MS TMT Analysis

The dried peptide samples for TMT analyses were reconstituted with 0.05% TFA in 2% acetonitrile and separated by a nanoflow LC system (Dionex UltiMate 3000 RSLC nano). Samples were injected onto a nanotrap column (Acclaim PepMap100 C18 Trap; 5 mm × 300 μm, 5 μm, 100 Å) at a flow rate of 25 μl/min for 3 min, using 0.1% formic acid (FA) in H_2_O. The following nano-LC gradient at 0.25 μl/min was used to separate the peptides: 0 to 10 min, 4 to 10% B; 10 to 75 min, 10 to 30% B; 75 to 80 min, 30 to 40% B; 80 to 85 min, 40 to 99% B; 85 to 89.8 min, 99% B; 89.8 to 90 min, 99 to 4% B; 90 to 120 min, 4% B; where A = 0.1% FA in H_2_O and B = 80% acetonitrile, 0.1% FA in H_2_O. The nanocolumn (EASY-Spray PepMap RSLC C18; 2 μm 100 Å, 75 μm × 50 cm) set at 40 °C was connected to an EASY-Spray ion source (Thermo Scientific). Spectra were collected from an Orbitrap mass analyzer (Orbitrap Fusion Lumos Tribrid; Thermo Scientific) using full MS mode (resolution of 120,000 at 400 *m*/*z*) over the *m*/*z* range 375 to 1500. Data-dependent MS2 scan was performed using quadrupole isolation in top speed mode using collision-induced dissociation activation and ion trap detection in each full MS scan with dynamic exclusion enabled. TMT reporter ion quantification was conducted using the multinotch MS3 method (13); the top5 most abundant MS2 fragment ions with an MS isolation window (*m*/*z*) of 0.7 were isolated, following higher-energy collisional dissociation activation and detection using the Orbitrap at a resolution of 60,000, generating an MS3 spectrum.

### DIA–MS Analysis

DIA–MS was conducted on a Q-Exactive HF (Thermo Scientific), using the above nanoflow-LC setup. All DIA samples were spiked with indexed retention time (iRT) peptide standards (Biognosys AG) to enable RT alignment. A precursor MS1 scan was conducted through the mass range of 400 to 1000 *m*/*z*, and MS2 spectra were collected in 25 *m*/*z*, nonoverlapping, isolation windows, in a step-wise manner throughout the mass range at a resolution of 30,000 (at 200 *m*/*z*). DIA data were analyzed in Spectronaut v11 (Biognosys AG) with a spectral library generated from data-dependent acquisition analyses of pooled control and LPS-treated human plasma samples, searched using Proteome Discoverer (version 2.2.0.388; Thermo Scientific) software with the parameters described later. MS1 and MS2 intensity extraction was set to maximum intensity, picking the highest data point within the *m*/*z* tolerance. MS1 and MS2 mass tolerance strategies were set to dynamic, whereby Spectronaut determines optimum tolerance based on extensive mass calibration. The precision iRT setting was selected within Spectronaut, utilizing a large set of calibration peptides to improve iRT precision and accuracy. Extracted ion chromatogram retention time extraction window was set to dynamic, dynamically adjusting the extracted ion chromatogram window in a retention time–dependent manner based on a large sample set during calibration. Precursor and protein *q*-value cutoff was set to 0.05. A minimum of three fragments were required for a peptide precursor to be added to the spectral library, up to a maximum of the six most abundant fragments. Quantification was conducted at an MS2 level, utilizing all fragment ion matches within the spectral library that pass the *Q*-value filter of 0.05.

### MS Database Search and Analysis

Proteome Discoverer software (version 2.2.0.388) was used to search raw data files against the human database (UniProtKB/Swiss-Prot version 2018_02; 20,400 protein entries) using Mascot (version 2.6.0; Matrix Science). The mass tolerance was set at 10 ppm for precursor ions and 0.8 Da for fragment ions. Trypsin was used as the digestion enzyme with up to two missed cleavages being allowed. Carbamidomethylation of cysteines and oxidation of methionine residues were chosen as fixed and variable modifications, respectively. Label-free quantification was conducted through the inbuilt Minora feature detection node, alongside RT alignment using the feature mapper node, with a maximum RT shift of 10 min being allowed. Unique and Razor peptide precursor areas were used for quantification, and cross-sample normalization was achieved using the total peptide amount. The inbuilt TMT 10-plex quantification method was assigned for detection of TMT labels. MS/MS-based peptide and protein identifications were validated with the following filters, a peptide probability of greater than 95.0% (as specified by the peptide prophet algorithm), a protein probability of greater than 99.0%, and at least two unique peptides per protein. Target false discovery rate (FDR) for peptide spectrum matches, peptide and protein identification was set to 1%. TMT-acquired data were again normalized using the total peptide amount. Data were then further scaled using the control pooled sample abundance, correcting for technical variation between injections and TMT groups.

### Immunoblotting

Laemmli sample buffer (4×) (62.5 mM Tris–HCl, pH 6.8, 10% glycerol, 1% SDS, 0.005% bromophenol blue, and 10% 2-mercaptoethanol) or the same buffer without 2-mercaptoethanol was mixed with protein samples and boiled at 95 °C for 10 min. Protein samples were separated using 4 to 12% Bis–Tris gradient gels (Thermo Scientific) in MOPS SDS running buffer (Thermo Scientific) at 130 V for 90 min. Gels were either stained for total protein using SimplyBlue Safe Stain (Thermo Fisher), or proteins were transferred onto nitrocellulose membranes in ice-cold transfer buffer (25 mM Tris base pH 8.3; 192 mM glycine; 20% methanol) at 350 mA for 2 h. Ponceau S red staining was used to determine efficient transfer and equal loading before membranes were blocked in 5% fat-free milk powder in PBS containing 0.1% Tween-20 (PBST; Sigma). Membranes were incubated in primary antibodies made to appropriate concentrations in 5% bovine serum albumin in PBST overnight at 4 °C. The membranes were then incubated in the appropriate light chain–specific peroxidase-conjugated secondary antibody in 5% milk/PBST. Antibody information is provided in supplemental Table S1. Membranes were washed for three times in PBST for 15 min before Western blots were developed using enhanced chemiluminescence (GE Healthcare) on photographic films (GE Healthcare). Densitometry analysis was done using the ImageJ analysis software.

### Whole-Blood RNA Quantification

Total RNA was extracted using the miRNeasy Mini kit (Qiagen) according to the manufacturer's recommendations. About 50 μl of whole blood was lysed in 700 μl of QIAzol reagent. Upon vigorous mixing and incubation, lysed cells, tissue, blood, or plasma were combined with 140 μl of chloroform, and the solution was mixed vigorously. Samples were then centrifuged at 12,000*g* for 15 min at 4 °C. About 280 μl of upper (aqueous) phase was carefully mixed with 420 μl of 100% ethanol and then applied to columns and washed according to the manufacturer's protocol. Total RNA was eluted in 35 μl of nuclease-free H_2_O by centrifugation at 9000*g* for 1 min at 4 °C. Concentration of cellular and tissue RNA was determined by spectrophotometry based on absorbance at 260 nm using NanoDrop 2000c (Thermo Scientific). For gene expression levels, reverse transcription was performed using the SuperScript VILO cDNA Synthesis Kit (Invitrogen). Per sample, 2 μl of VILO RT Master Mix were combined with 8 μl of sample in a 25 to 100 ng/μl dilution. Thermal cycler stages were set as follows: incubation at 25 °C for 10 min and 42 °C for 120 min, followed by termination of the reaction at 85 °C for 5 min. RT-PCR and preamplification products were stored at −80 °C. TaqMan assays were used to assess the relative gene expression levels (supplemental Table S2). About 2.25 μl of diluted RT product was combined with 0.25 μl of TaqMan assay (20×) (Applied Biosystems) and 2.5 μl of the TaqMan Universal PCR Master Mix 11 No AmpErase UNG (2×) to a final volume of 5 μl. Reactions were loaded using a Bravo Automated Liquid Handling Platform (Agilent). Quantitative PCR was performed on a ViiA7 RealTime PCR System (Applied Biosystems) at 95 °C for 10 min, followed by 40 cycles of 95 °C for 15 s and 60 °C for 1 min. Relative quantification was performed using the 2-''Cq method with proprietary Viia7 software (Applied Biosystems).

### Statistical Analysis

Statistical analyses were conducted using the open-source software Perseus (14). Proteomic datasets were filtered to keep only molecules with less than 30% missing values in one experimental group. The remaining missing values were imputed from a normal distribution. The relative quantities of proteins were scaled using the inbuilt *z*-score function. Fold differences represented within all volcano plots were obtained after *z* scoring. Multiple sample testing was conducted by ANOVA with permutation-based FDR correction, whereas two-sample comparisons were made using the Student's *t* test again with permutation-based FDR correction with a threshold of 5%. Hierarchical clustering distance was set to Euclidean, and the linkage method used was the average. All other data visualizations were created in GraphPad Prism (version 8.2.0; GraphPad Software). Schematic diagrams were created using BioRender (Biorender.com).

## Results

### The Effect of Low-Dose Endotoxemia on the Human Plasma Proteome

**Fig. 1:**
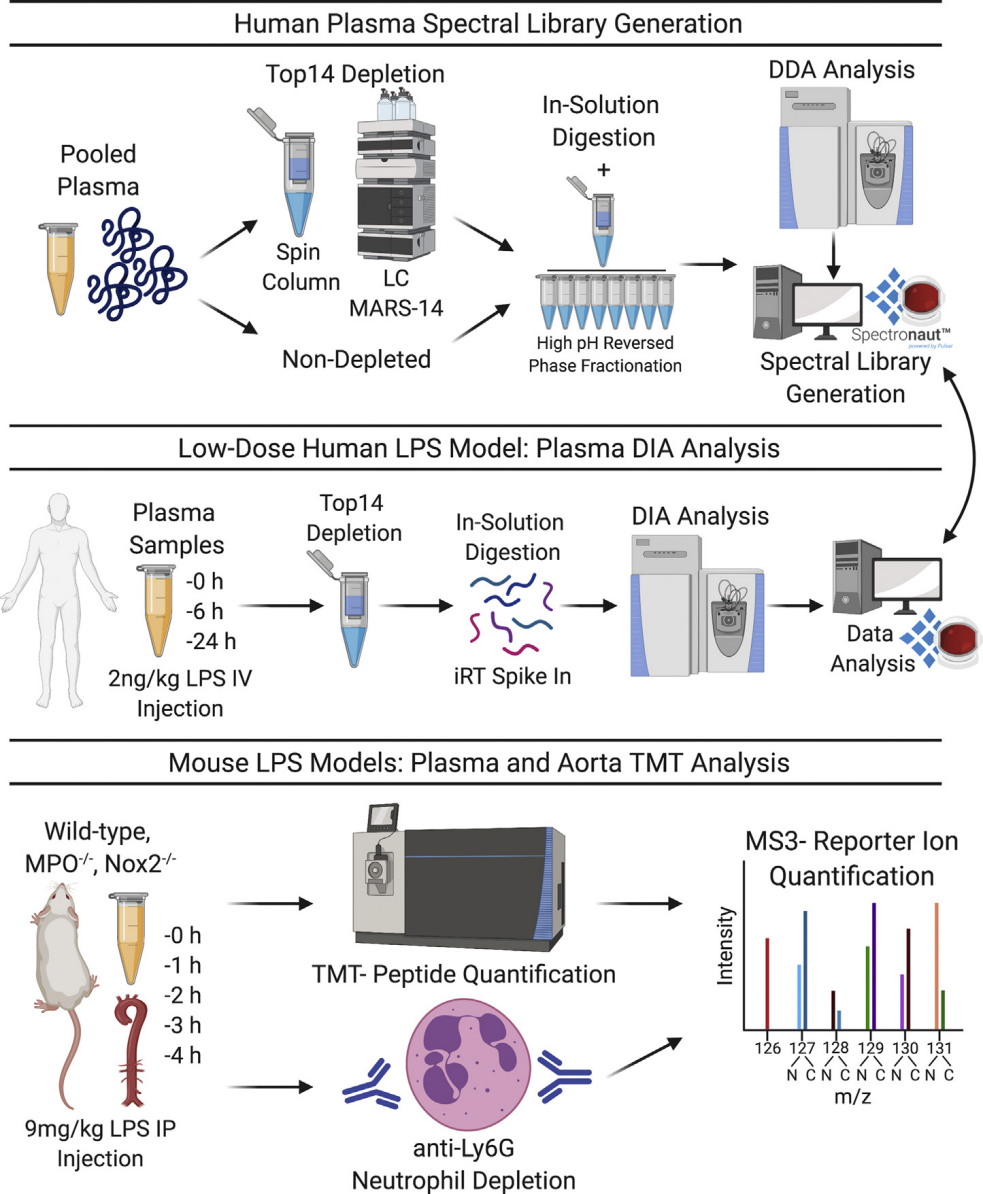
**Overview of experiments**. DIA–MS was performed in serial plasma samples from human volunteers who received low-dose endotoxin. TMT-based quantitation was used for plasma, and aortic samples obtained from a murine endotoxemia model. DDA, data-dependent acquisition; DIA, data-independent acquisition; iRT, indexed retention time; LPS, lipopolysaccharide; MPO, myeloperoxidase; Nox2, NADPH oxidase 2; TMT, tandem mass tag.

**Fig. 2:**
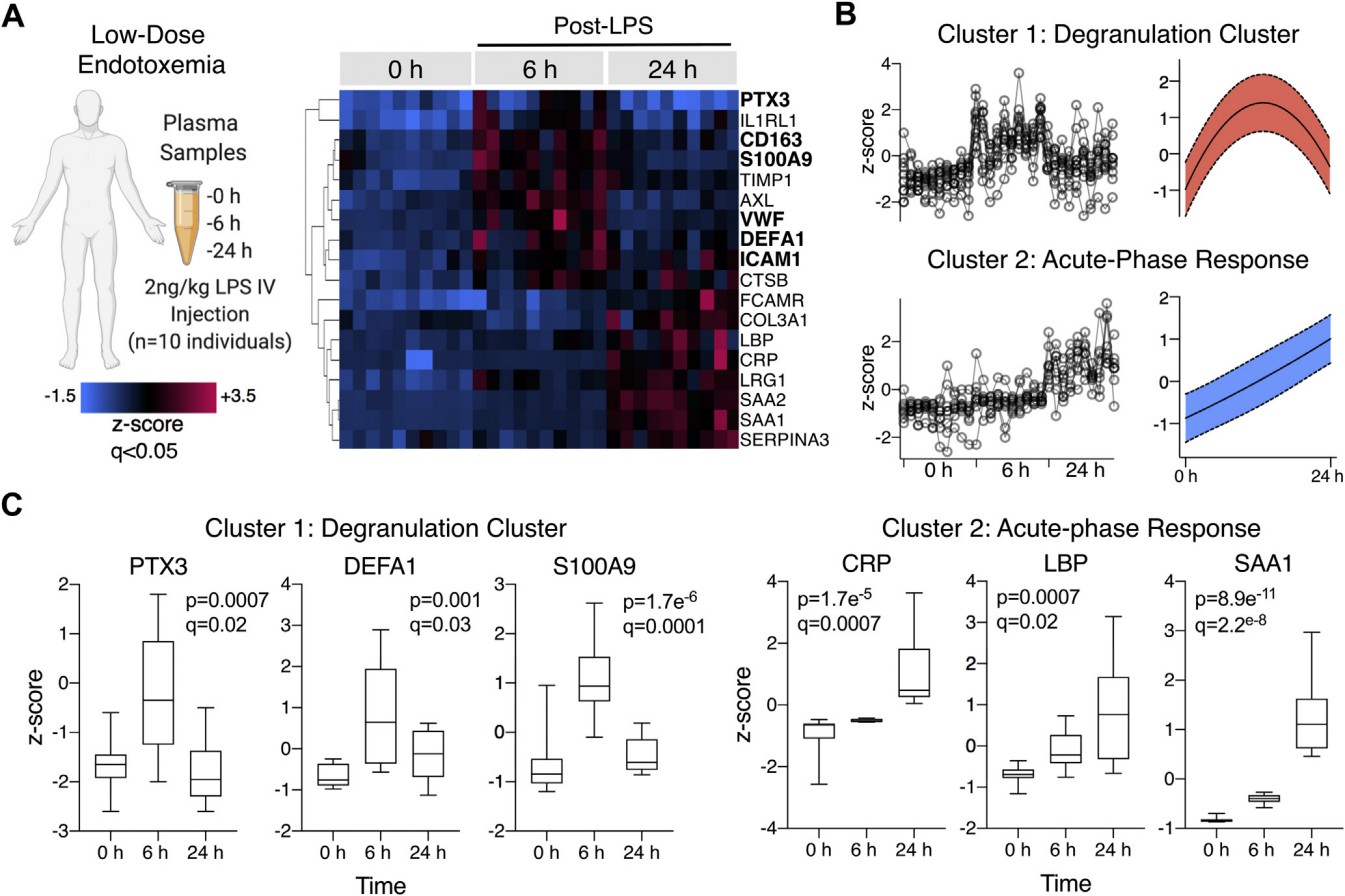
**The effect of low-dose endotoxemia on the human plasma proteome as revealed by data-independent acquisition MS (DIA–MS).***A*, healthy human volunteers were injected with low-dose endotoxin (*n* = 10; LPS 2 ng/kg, i.v.). Plasma isolated at 0, 6, and 24 h after LPS injection was analyzed by DIA–MS. Hierarchical cluster analysis of significantly changing proteins (*q* < 0.05) over the LPS time course is represented in a heat map. *B*, graphical representation of two clusters of protein changes over the LPS time course. *C*, core protein members of the degranulation and acute phase responses are shown in box plots. Significance was determined by ANOVA with false discovery rate–based correction for multiple testing. LPS, lipopolysaccharide.

Healthy volunteers were challenged with low-dose endotoxin (LPS; 2 ng/kg, *i.e.*, *n* = 10 individuals) (Fig. 1) (12). Volunteer characteristics are provided in supplemental Table S3. DIA–MS was utilized to map the protein response in serial plasma samples over three time points. Initially, a human plasma spectral library was generated through the data-dependent acquisition analysis of pooled samples either nondepleted, top2-depleted, or top14-depleted and high-pH reverse phase peptide fractionated, enabling the confident identification of 712 proteins with a minimum of two unique peptides (Fig. 1). DIA was then conducted upon top14-depleted plasma samples from the human LPS cohort, enabling the identification and quantification of 608 proteins. Hierarchical cluster analysis of significantly changing proteins in human plasma over the 24 h LPS time course revealed two distinct protein clusters (Fig. 2, *A* and *B*). A protein degranulation response was evident, whereby proteins of neutrophil (PTX3, S100 calcium-binding protein A9—S100A9, and neutrophil defensin 1—DEFA1) and platelet or endothelial origin (von Willebrand factor) were transiently increased at 6 h post-LPS injection before returning to baseline at 24 h (cluster 1; Fig. 2*B*). Furthermore, a global correlation between all proteins identified in human plasma identified S100A9 and DEFA1 as the two strongest protein correlates to PTX3 (Pearson coefficient = 0.6 and *p* < 0.001, for both interactions). A later liver-driven response was observed, where acute phase reactants were highest in abundance at 24 h post-LPS injection (cluster 2; Fig. 2*B*), including C-reactive protein (CRP), serum amyloid A-1 (SAA1), serum amyloid A-2 (SAA2), and lipopolysaccharide-binding protein (LBP) (Fig. 2*A*). Core proteins of the degranulation and acute phase responses are individually represented, highlighting the temporal PTX3 response over the LPS time course (Fig. 2*C*). Two-group comparison volcano plots (0 h *versus* 6 h, 0 h *versus* 24 h and 6 h *versus* 24 h) highlight other important plasma protein changes over time. Protein changes only observed at 6 h after LPS administration included the reduction in the von Willebrand factor–cleaving protease, a disintegrin and metalloproteinase with a thrombospondin type-1 motif member 13 (ADAMTS13) as well as the rise in the monocyte/macrophage marker CD163, tyrosine protein kinase receptor UFO (AXL), and S100A9 (supplemental Fig. S3). All significant changes over the LPS time course in human plasma are provided in supplemental Table S4.

### Confirmation in a Murine Endotoxemia Model

**Fig. 3:**
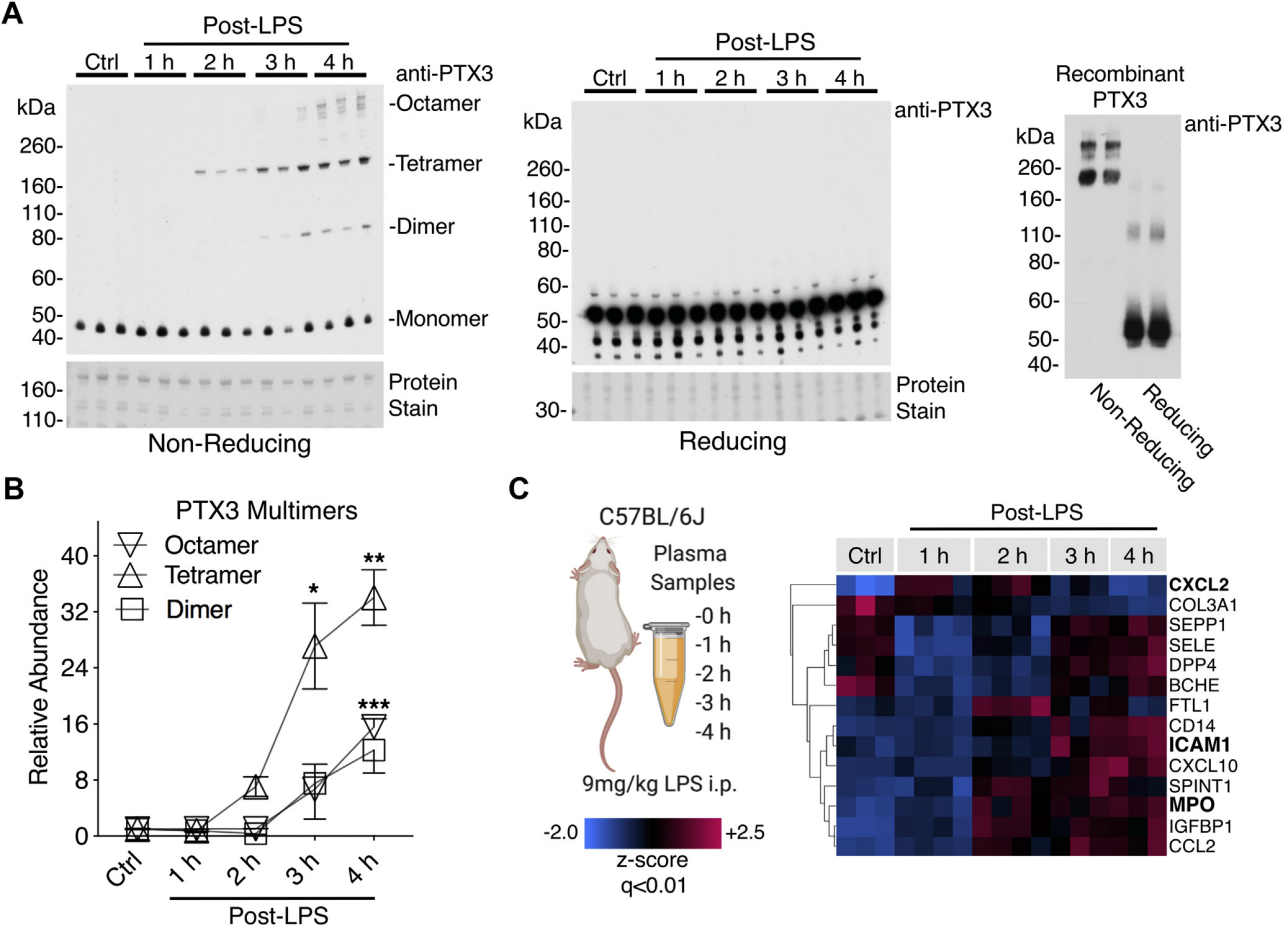
**Confirmation in a murine endotoxemia model.***A*, plasma isolated from mice injected with LPS (9 mg/kg, i.p.) was analyzed by immunoblotting under both non-reducing and reducing conditions (ctrl, 1, 2, 3, and 4 h, *n* = 3 per time point). Recombinant PTX3 was also separately analyzed by immunoblotting under both non-reducing and reducing conditions. *B*, densitometry analysis of PTX3 multimers over the LPS time course. *C*, murine plasma was depleted of the top seven most abundant proteins and analyzed by TMT–based quantitative MS. A hierarchical cluster analysis of significantly changing proteins (*q* < 0.01) is presented as a heat map. Significance was determined by ANOVA with false discovery rate–based correction for multiple testing. LPS, lipopolysaccharide; PTX3, pentraxin-3; TMT, tandem mass tag.

We have previously shown that the redox state of PTX3 and its oligomeric composition may serve as a marker of resolution of inflammation in patients with sepsis (11). An increase in PTX3 was part of the degranulation response observed in human plasma 6 h post-LPS injection alongside neutrophil-derived proteins such as S100A9 and DEFA1 (Fig. 2*C*). To further explore the kinetics of this PTX3 response, we adopted an LPS endotoxemic mouse model (Fig. 1). Mice were injected with LPS (9 mg/kg, i.p.) and harvested at hourly time points (1, 2, 3, and 4 h), whereas control mice were injected with saline. Western blot analysis of murine plasma confirmed a rapid time-dependent increase in the abundance of multimeric PTX3 in response to LPS injection (Fig. 3*A*, *left panel*). The multimers of PTX3 are dependent upon the formation of interdisulfide bonds, as PTX3 multimers are undetectable under reducing conditions (Fig. 3*A*, *middle panel*) (8). Recombinant PTX3 was used to confirm the multimeric distribution of PTX3 by immunoblotting (Fig. 3*A*, *right panel*). Densitometry analysis revealed dimeric, tetrameric, and octameric forms of PTX3 to be increased in murine plasma post LPS and undetectable in controls (Fig. 3*B*). Analogous to our analysis of human plasma, we utilized multiplexed proteomics to determine changes within mouse plasma in response to endotoxemia. Mouse plasma was depleted of the top7 most abundant proteins and analyzed by TMT-based MS. C–X–C motif chemokine 2 (CXCL2), a powerful neutrophil chemoattractant (15), was among the earliest detectable protein changes within 1 h after LPS injection. In contrast to the low-dose endotoxemia experiment in humans, PTX3 was not detected by MS in mouse plasma, probably because of the differences in depletion and MS methods (top7 *versus* top14 depletion and TMT–MS *versus* DIA–MS [Fig. 3*C*]). Consistent with our findings in humans, plasma levels of intercellular adhesion molecule 1 (ICAM-1) were increased (Fig. 3*C*). The neutrophil-derived oxidant-generating enzyme, MPO, preceded the rise in cell–cell adhesion molecules (Fig. 3*C*). Gene Ontology enrichment analyses upon proteins changing with nominal significance (*p* < 0.05) over the LPS time course in humans and mice are compared in supplemental Figure S4. Significant plasma protein changes over the mouse LPS time course are provided in supplemental Table S5.

### MPO and Nox2 Are Not Required for PTX3 Multimer Formation

**Fig. 4:**
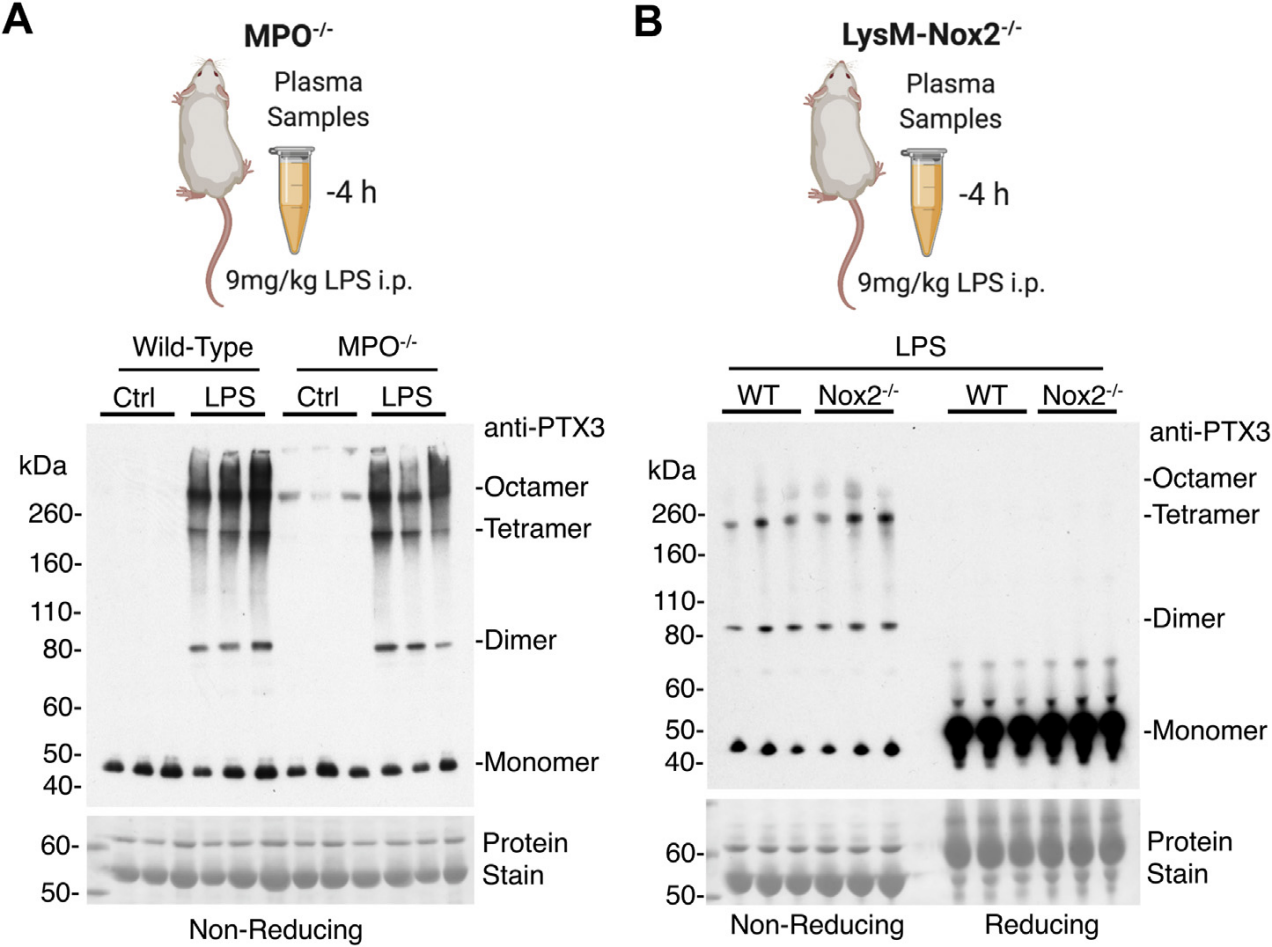
**MPO and Nox2 are not required for PTX3 multimer formation.***A*, plasma isolated from MPO KO mice was used to determine the requirement of this oxidant-generating enzyme in the formation of multimeric PTX3. *B*, similarly, mice with Nox2 deficiency in myeloid cells (LysM-Nox2−/−) were treated with LPS, and plasma was assessed by immunoblotting. LPS, lipopolysaccharide; MPO, myeloperoxidase; Nox2; NADPH oxidase; PTX3, pentraxin-3.

The rise in plasma MPO after LPS injection in mice coincided with the increase in multimeric forms of PTX3, where PTX3 tetramers were also first observed 2 h post-LPS injection (Fig. 3, *A*–*C*). To determine whether MPO was the oxidant-generating enzyme responsible for the formation of multimeric PTX3 in response to LPS, MPO–KO mice were utilized. Multimeric PTX3 was still observed in the plasma of MPO^−/−^ mice treated with LPS (Fig. 4*A*). Another prominent oxidant-generating enzyme key to the inflammatory response is neutrophil Nox2, responsible for the generation of reactive oxygen species during the respiratory burst (16). However, plasma of mice lacking Nox2 specifically in myeloid cells (17) had similar levels of multimeric PTX3 post-LPS injection, as wildtype mice (Fig. 4*B*). Thus, PTX3 multimers are not dependent on the two key reactive oxygen species-generating enzymes and appear to be stored and released rather than formed during neutrophil activation.

### Vascular Deposition of PTX3 and HDL-Associated Apolipoproteins

**Fig. 5:**
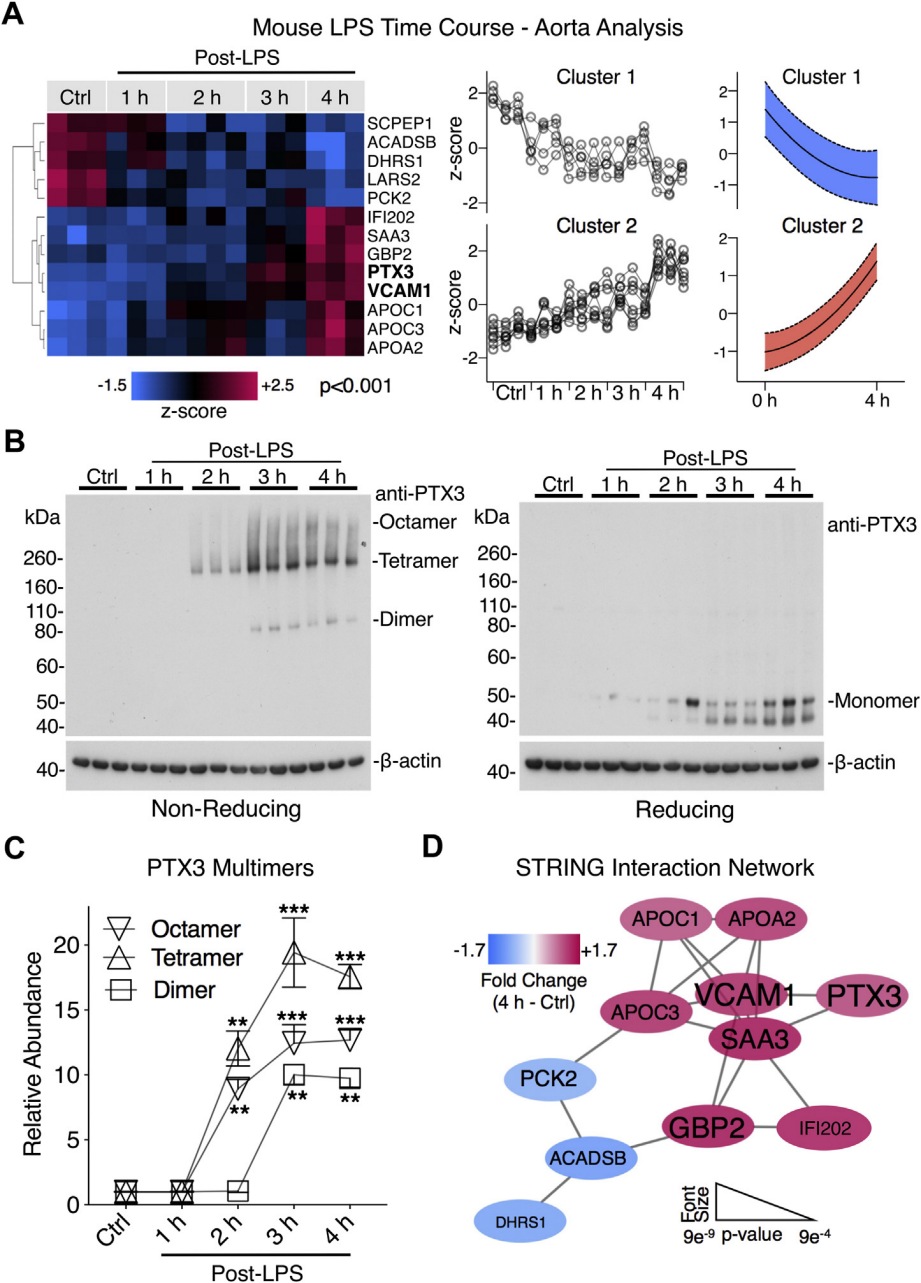
**Vascular deposition of PTX3 and high-density lipoprotein–associated apolipoproteins.***A*, aortic protein extracts isolated from mice injected with LPS (9 mg/kg, i.p.) were quantified by TMT–based MS (ctrl, 1, 2, 3, and 4 h, *n* = 3 or 4 per time point). A hierarchical cluster analysis of significantly changing proteins (*p* < 0.001) is shown as a heat map. Two clusters of protein changes within the aorta over the LPS time course are graphically represented. *B*, the aortic PTX3 response was confirmed by immunoblotting, under both non-reducing and reducing conditions. *C*, densitometry analysis of PTX3 multimers over the LPS time course. *D*, STRING interaction network between the significantly changing proteins within the aorta over the LPS time course. Font size relates to significance level, whereas color represents directionality of change. Significance was determined by ANOVA with false discovery rate–based correction for multiple testing. LPS, lipopolysaccharide; PTX3, pentraxin-3; TMT, tandem mass tag.

Leukocyte extravasation is a key feature of sepsis. To assess the time course of vascular protein changes in the endotoxemia model, aortas were harvested from C57BL/6J wildtype mice and analyzed by multiplexed proteomics (baseline, 1, 2, 3, and 4 h after LPS administration). Hierarchical cluster analysis of significantly changing proteins (*p* < 0.001) in the aorta over the 4 h LPS time course revealed two distinct protein clusters (Fig. 5*A*). Notably, PTX3 was among the most upregulated proteins within the aorta at 4 h post LPS, mirroring increases in interferon-achievable protein 202 (IFI202), vascular cell adhesion molecule-1 (VCAM-1), and high-density lipoprotein (HDL)–associated apolipoproteins, including apolipoprotein-A2 (ApoA2) (cluster 2; Fig. 5*A*). Reduced proteins within the aorta post-LPS injection were primarily involved in metabolic processes, including phosphoenolpyruvate carboxykinase (PCK2, an enzyme that catalyzes the rate-limiting step of gluconeogenesis) (cluster 1; Fig. 5*A*). Significant aortic protein changes over the mouse LPS time course are provided in supplemental Table S6. Western blot analysis confirmed the deposition of multimeric PTX3 within the aorta, as early as 2 h post-LPS injection (Fig. 5, *B* and *C*). In contrast, PTX3 was undetectable in aortic tissue from saline-injected control mice (Fig. 5*B*). Time-course analysis of PTX3 within the aorta revealed high PTX3 multimer levels at 3 h post-LPS injection (Fig. 5*C*). A STRING interaction network generated upon significantly changing proteins in the aorta highlights a relationship between PTX3 and VCAM-1 in response to LPS (Fig. 5*D*). This is notable since a core feature of both human and mouse plasma responses to LPS was the evident increase in proteins related to cell–cell adhesion, the most prominent of which being ICAM-1 (Figs. 2*A* and 3*C*).

### Neutrophils Contribute to PTX3 Deposition Within the Vasculature

**Fig. 6:**
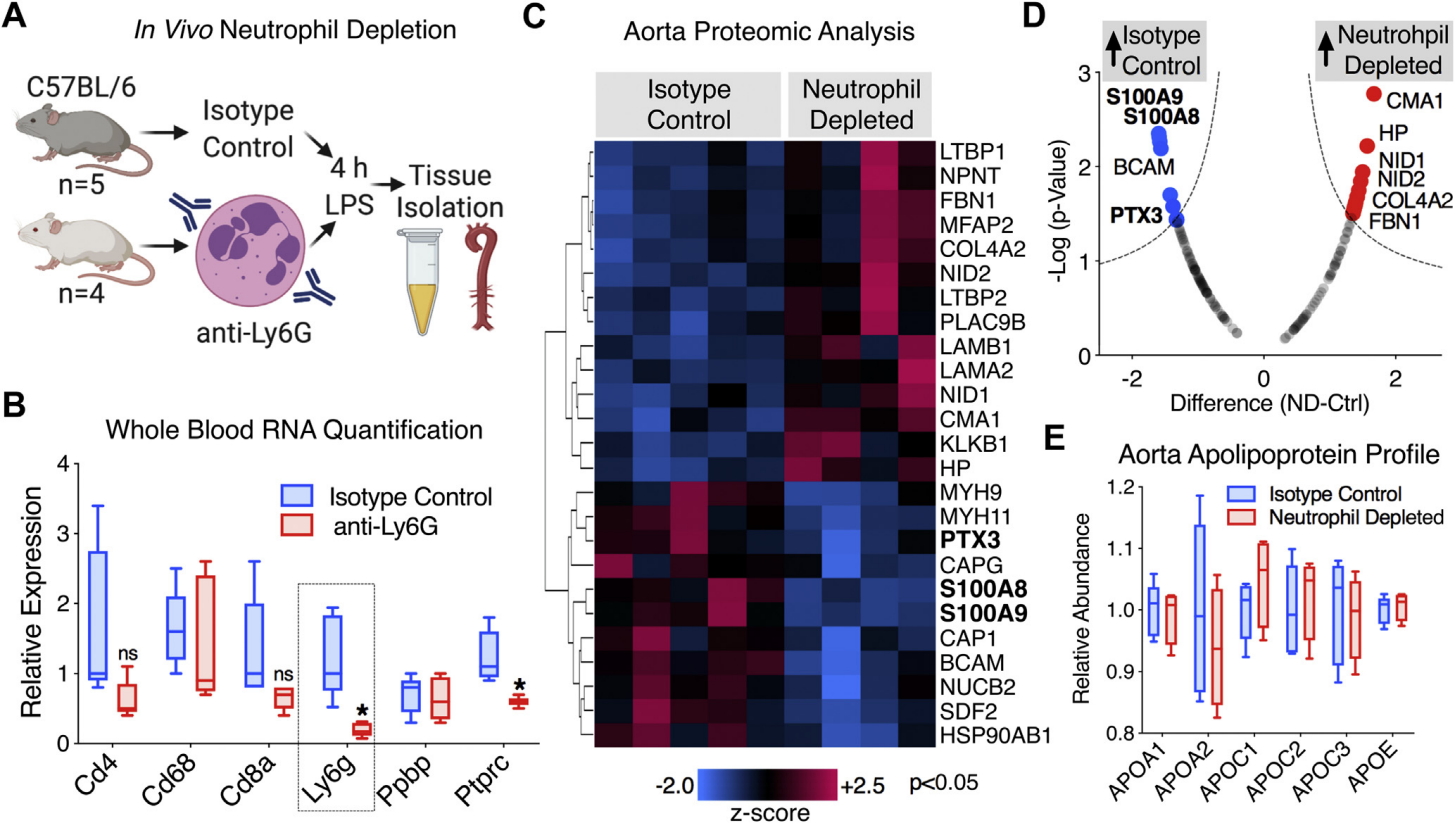
**Neutrophils contribute to PTX3 deposition within the vasculature.***A*, wildtype mice were depleted of neutrophils by anti-Ly6G antibody injection (i.p.) 20 h prior to LPS treatment (9 mg/kg, 4 h); control mice were treated with isotype control antibody (*n* = 5). *B*, whole-blood RNA quantification was used to determine efficient and specific depletion of neutrophils in the circulation. *C*, aortic protein extracts were analyzed by TMT–based quantitative MS. Heat map displays hierarchical clusters of significantly changing proteins (*p* < 0.05) between neutrophil-depleted and isotype control mice. *D*, a volcano plot depicting aortic protein changes between neutrophil-depleted and isotype control mice is shown. The MS dataset was filtered to retain proteins of known extracellular localization. *E*, high-density lipoprotein–associated apolipoproteins within the aorta showed no change. Significance was determined by *t* test with false discovery rate–based correction for multiple testing. LPS, lipopolysaccharide; PTX3, pentraxin-3; TMT, tandem mass tag.

The rapid deposition of multimeric PTX3 within the aorta, 2 h post-LPS injection, suggests cell infiltration rather than PTX3 *de novo* synthesis within the vasculature, further corroborated by the fact that PTX3 was undetectable in control aortic tissue. Second, the PTX3 response to endotoxemia consistently correlated closely to changes in cell adhesion molecules. To determine to what extent neutrophils deposit PTX3 within the vasculature, mice were treated with an anti-Ly6G antibody (Fig. 6*A*). Successful depletion of neutrophils was confirmed through whole-blood RNA analysis (Fig. 6*B*). Hierarchical cluster analysis of significantly changing proteins (*p* < 0.05) in the aorta after LPS injection revealed two distinct protein clusters (Fig. 6*C*). LPS-treated mice depleted of neutrophils showed reduced deposition of PTX3, S100A8, and S100A9 within the aorta compared with isotype-treated control mice (Fig. 6*D*). In contrast, many extracellular matrix (ECM) proteins were increased in abundance in aortic extracts from LPS-treated neutrophil-depleted mice, when compared with isotype control mice, including nidogen-1 and 2, collagen type IV, alpha 2, and fibrilin-1 (Fig. 6*D*), reflecting the high ECM-degrading potential of infiltrating neutrophils. Significant aortic protein changes as a result of neutrophil depletion and LPS treatment are provided in supplemental Table S7. To rule out that LPS-induced changes in vascular permeability rather than leukocyte infiltration contribute to PTX3 deposition within the aorta (18), we compared the increase in HDL-associated apolipoproteins, such as ApoA2, apolipoprotein-C1, and apolipoprotein-C2 (Fig. 5*A*). Among lipoproteins, HDL is most likely to increase with higher vascular permeability because of its nanoparticle size. Yet, neutrophil depletion had no influence upon apolipoprotein levels within the aorta of LPS-treated mice (Fig. 6*E*).

## Discussion

The present study includes in-depth proteomic analyses of the endotoxemia response in humans and mice. DIA–MS analysis from plasma derived from healthy volunteers with low-dose endotoxemia revealed two distinct clusters of protein changes: an initial degranulation response containing PTX3, followed by an acute phase response, including CRP, LBP, SAA1, and SAA2. The degranulation response was further interrogated by proteomics in a murine model of endotoxemia. The earliest detectable change after LPS injection in mice was a rise in CXCL2, a potent neutrophil chemoattractant. This was followed by a neutrophil degranulation response as evidenced by the rise in MPO and PTX3. *In vivo* depletion experiments confirmed that neutrophils are predominantly responsible for the vascular deposition of PTX3 alongside calprotectin, a complex of S100A8 and S100A9 that constitutes about half of the total protein content in the neutrophil cytosol.

Studies that interrogate the human plasma proteome after a defined inflammatory stimulus are limited (19). Furthermore, the extent to which certain cell types and organs contribute to temporal plasma proteome changes during the inflammatory response is currently poorly defined (20). We therefore analyzed the plasma proteome of healthy volunteers injected with low-dose LPS, utilizing depletion technologies for abundant plasma proteins and DIA–MS. Our DIA workflow enabled the quantification of 608 proteins in human plasma, which included 59 Food and Drug Administration–approved protein biomarkers (21). A rapid degranulation response was observed 6 h post-LPS injection, followed by a later acute phase response with primarily liver-derived plasma proteins. PTX3 followed similar kinetics to other proteins primarily known to be secreted by neutrophils, including S100A9 and DEFA1 (22, 23). Furthermore, CD163, a monocyte-macrophage marker, also followed a similar profile to that of PTX3. CD163 is a known biomarker in settings of acute inflammation and is shed by the inflammation-responsive protease disinterring and metalloproteinase domain–containing protein 17 (ADAM17/TACE) (24). CD163 negatively correlates with sepsis survival (25).

In a previous work by Calvano *et al*. (26), transcriptomic profiling was conducted upon whole blood leukocytes in humans using the same endotoxin and dose as in our study. Rather than focusing on the transcriptional response in blood leukocytes, our analysis of the human plasma proteome allowed us to quantify the contribution of other organs, such as the liver acute phase response as evidenced by elevated plasma levels of SAA1, SAA2, LBP, and CRP at 24 h after LPS administration. Interestingly, the study by Calvano *et al*. (26) identified an increase in a cluster of protein changes related to the superoxide-generating NADPH oxidase system, suggesting a net increase in oxidant generation over the immune response that could contribute to protein disulfide bond formation and protein multimerization. With regard to PTX3, however, myeloid Nox2-deficient mice had detectable PTX3 multimers. Thus, the formation of the protein disulfide bonds in PTX3 is not dependent on the NADPH oxidase–driven respiratory burst in neutrophils.

The degranulation response observed in human plasma was confirmed in an endotoxemic mouse model, whereby the oxidant-generating enzyme MPO correlated closely with the detection of PTX3 multimers in plasma. MPO is a key constituent of the neutrophil cytotoxic response to invading pathogen, being released from azurophilic granules to catalyze the production of the bactericidal oxidant, hypochlorous acid (27). Critically, PTX3 is also known to be stored within azurophilic granules of the neutrophil and therefore led us to hypothesize that MPO could be responsible for the oxidation and subsequent formation of PTX3 multimers in response to LPS (28). Jaylon *et al*. (28) have previously reported that monomeric PTX3 is detectable within the neutrophil and that, upon stimulation, multimeric PTX3 is detected within cell supernatants, suggesting that an active oxidation of PTX3 is occurring upon neutrophil stimulation. However, mice deficient of MPO have no change in multimeric PTX3 in response to LPS injection when compared with wildtype animals, demonstrating that this oxidant-generating enzyme is also not required for PTX3 multimerization. Thus, PTX3 may already be stored in its multimeric form.

Another common feature of both the responses to LPS in humans and mice was the presence of cell–cell adhesion proteins within the circulation, most prominently ICAM-1. ICAM-1 forms a critical bridge between the endothelium and circulating leukocytes, whereby ICAM-1 promotes the slow rolling and subsequent infiltration of leukocytes, particularly neutrophils, to the subendothelial space. Furthermore, soluble ICAM-1 alongside other circulating adhesion molecules such as the selectins that we also observe to be modulated by LPS injection are markers of vascular inflammation (29, 30). The rise in circulating adhesion molecules suggests an activated endothelium as origin of adhesion molecule shedding (30). Interestingly, recent reports have shed light on the role that MPO plays in the promotion of neutrophil infiltration into the vasculature (31, 32, 33).

Multiplexed vascular proteomics revealed PTX3 to be the most significantly increased protein within the aorta, whereby PTX3 tetramers were present as early as 2 h post-LPS injection, a rapidity that is consistent with protein deposition rather than *de novo* synthesis. This deposition of PTX3 within the aorta was again accompanied by a rise in an adhesion molecule, VCAM-1, as well an increase in HDL-associated apolipoproteins, including ApoA2. HDL levels rapidly decline in patients with sepsis, and the extent of this decrease is associated with a poor prognosis (34). Since endotoxemia increases vascular permeability and HDL is the smallest among all lipoproteins, LPS will facilitate leakage of HDL from the lumen of the blood vessels (35). Finally, the *in vivo* depletion of neutrophils in mice treated with LPS attenuated PTX3 deposition within the aorta, along with a reduction in neutrophil-derived calprotectin (S100A8 and S100A9). A concomitant increase in many ECM proteins, including core constituents of the endothelial basement membrane such as nidogen-1, collagen type IV, alpha 2, and fibrilin-1, highlights the known role of the neutrophil in matrix degradation (36).

In conclusion, using a wide array of proteomic methodologies in both human and mouse models, we elucidated a distinct relationship between PTX3 and neutrophils in the context of endotoxemia and associated vascular inflammation. DIA–MS during low-dose endotoxemia in healthy volunteers highlighted the temporal sequence of the human plasma proteome response, with protein degranulation being an initial core feature, which included PTX3. Multiplexed proteomics in mouse plasma and aortic tissue revealed the deposition of PTX3 multimers in the vessel wall. Neither MPO nor Nox2, as major oxidant-generating enzymes in neutrophils, is required for PTX3 multimerization. Instead, neutrophil extravasation accounts for the vascular deposition of PTX3 multimers during endotoxemia.

## Data availability

The MS proteomics data have been deposited to the ProteomeXchange Consortium (http://proteomecentral.proteomexchange.org) via the PRIDE partner repository (13) with the dataset identifier <PXD021077>.

## Conflict of interest

King's College London has filed and licensed a patent application with regard to using PTX3 multimers as a biomarker in sepsis. R. F. S. reports research grants and personal fees from AstraZeneca, Cytosorbents, GlyCardial Diagnostics, and Thromboserin and personal fees from Bayer, Bristol Myers Squibb/Pfizer, Hengrui, Idorsia, Intas Pharmaceuticals, PhaseBio, Portola, and Sanofi Aventis.
