# Supplementary material for: A Proteomics-Based Assessment of Inflammation Signatures in Endotoxemia
Source: Mol Cell Proteomics. 2021 Feb 24;20:100021. doi: 10.1074/mcp.RA120.002305 (PMC7950208; doi:10.1074/mcp.RA120.002305)
Supplement: Supplemental Figures S1 to S4 and Supplemental Tables S1 to S7 [file mmc1.pdf]

SUPPLEMENTAL DATA:

A Proteomics-based Assessment of Inflammation Signatures in Endotoxemia

Sean A. Burnap[1], Ursula Mayr[1], Manu Shankar-Hari[2], Friederike Cuello[3], Mark R. Thomas[4], Ajay M. Shah[1], Ian Sabroe[5], Robert F. Storey[4], Manuel Mayr[1]*.

Running title: Plasma proteome response to endotoxin

*To whom correspondence should be addressed:

Manuel Mayr; King's British Heart Foundation Centre, King's College London, 125 Coldharbour Lane, London, SE59NU UK;

Phone: +44(0)2078485446; +44(0)2078485298; Email: manuel.mayr@kcl.ac.uk

Supplemental Figures

Supplemental Figure 1. An assessment of analytical reliability.

Supplemental Figure 2. An assessment of reproducibility.

Supplemental Figure 3. The effect of low-dose endotoxemia on the human plasma proteome

Supplemental Figure 4. A comparison between human and mouse plasma proteome responses to LPS

Supplemental Tables

Supplemental Table S1. Immunoblot antibody information

Supplemental Table S2. TaqMan assays for qPCR

Supplemental Table S3. Baseline characteristics of healthy volunteers

Supplemental Table S4. Protein changes in DIA analysis of human plasma during the LPS time course

Supplemental Table S5. Protein changes in TMT analysis of mouse plasma during the LPS time course

Supplemental Table S6. Protein changes in TMT analysis of murine aortas during the LPS time course

Supplemental Table S7. Protein changes in TMT analysis of murine aortas after neutrophil depletion

Supplemental Excel Files

Supplemental Table S8. Human plasma LPS time course DIA Spectronaut export

Supplemental Table S9. Mouse plasma LPS time course TMT Proteome Discoverer export

Supplemental Table S10. Mouse aorta LPS time course TMT Proteome Discoverer export

Supplemental Table S11. Mouse aorta LPS Neutrophil Depleted TMT Proteome Discoverer export

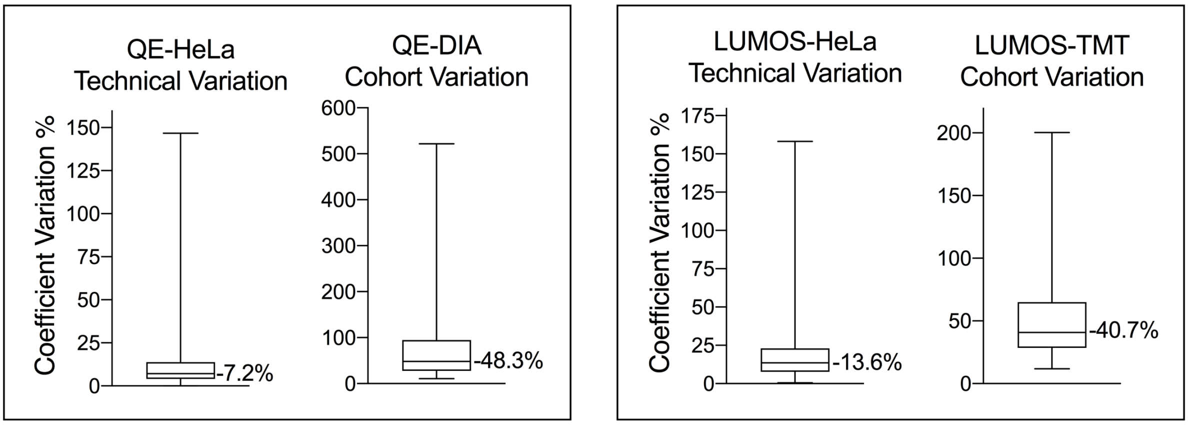

Supplemental Fig 1. ***An assessment of analytical reliability***. A standard HeLa protein digest was injected at regular intervals across cohort analyses on both the Q Exactive HF (***left panel***), utilised for DIA analysis, and the Fusion Lumos (***right panel***), used for TMT analysis. The coefficient of variation (CV%) of protein abundances across triplicate HeLa injections was determined and the median protein variation is shown. Protein abundance variation in the cohorts analysed are shown alongside, highlighting that biological variation is greater than that observed for technical variation.

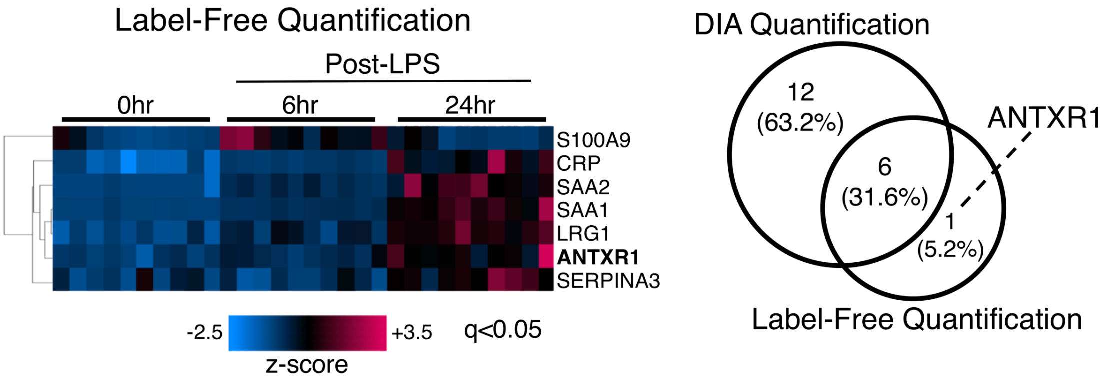

Supplemental Fig 2. *An assessment of reproducibility*. Human LPS samples that were top14 depleted and analysed by DIA-MS were also analysed by label-free quantification in a non-depleted form. Significantly (q<0.05) changing proteins over time are represented as a heat map. The rise in acute phase proteins, representing more abundant plasma proteins, observed to be changing by DIA-MS were also validated by label-free quantification. Further highlighting the strength of DIA-MS and protein depletion in the detection of lower abundant plasma proteins.

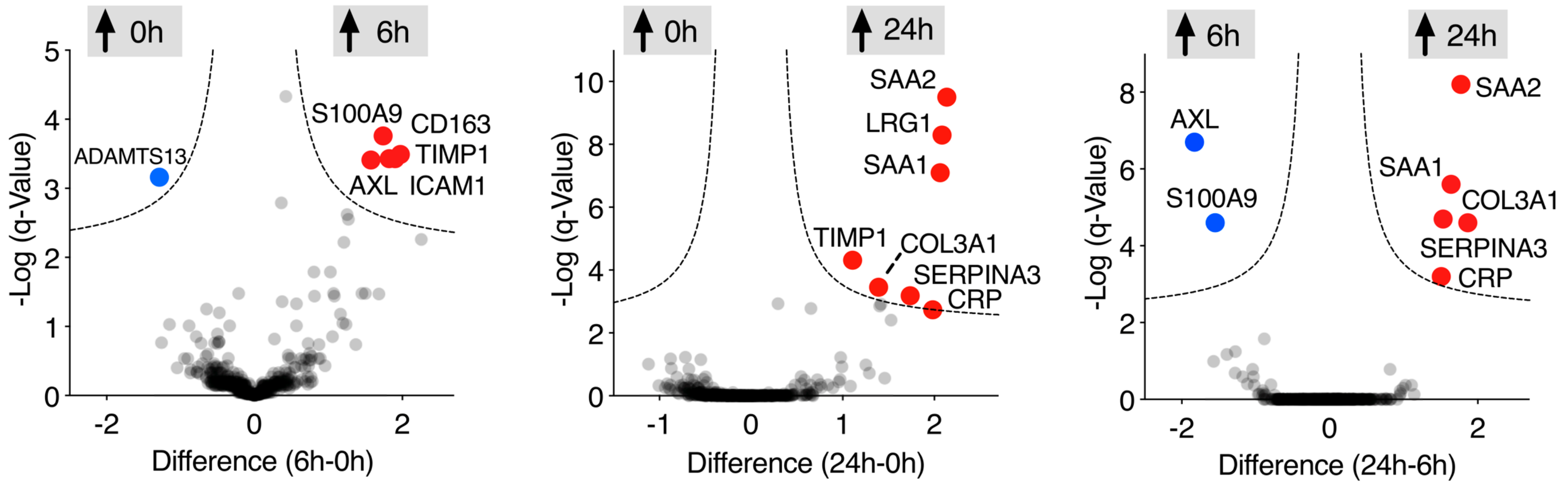

Supplemental Fig 3. ***The effect of low-dose endotoxemia on the human plasma proteome.*** Healthy human volunteers were injected with low-dose endotoxin (n=10, LPS 2 ng/kg, i.v.). Plasma isolated at 0, 6 and 24 h post LPS injection was analysed by DIA-MS. Volcano plots show corresponding two group comparisons. Significance was determined by t-tests with FDR-based correction for multiple testing.

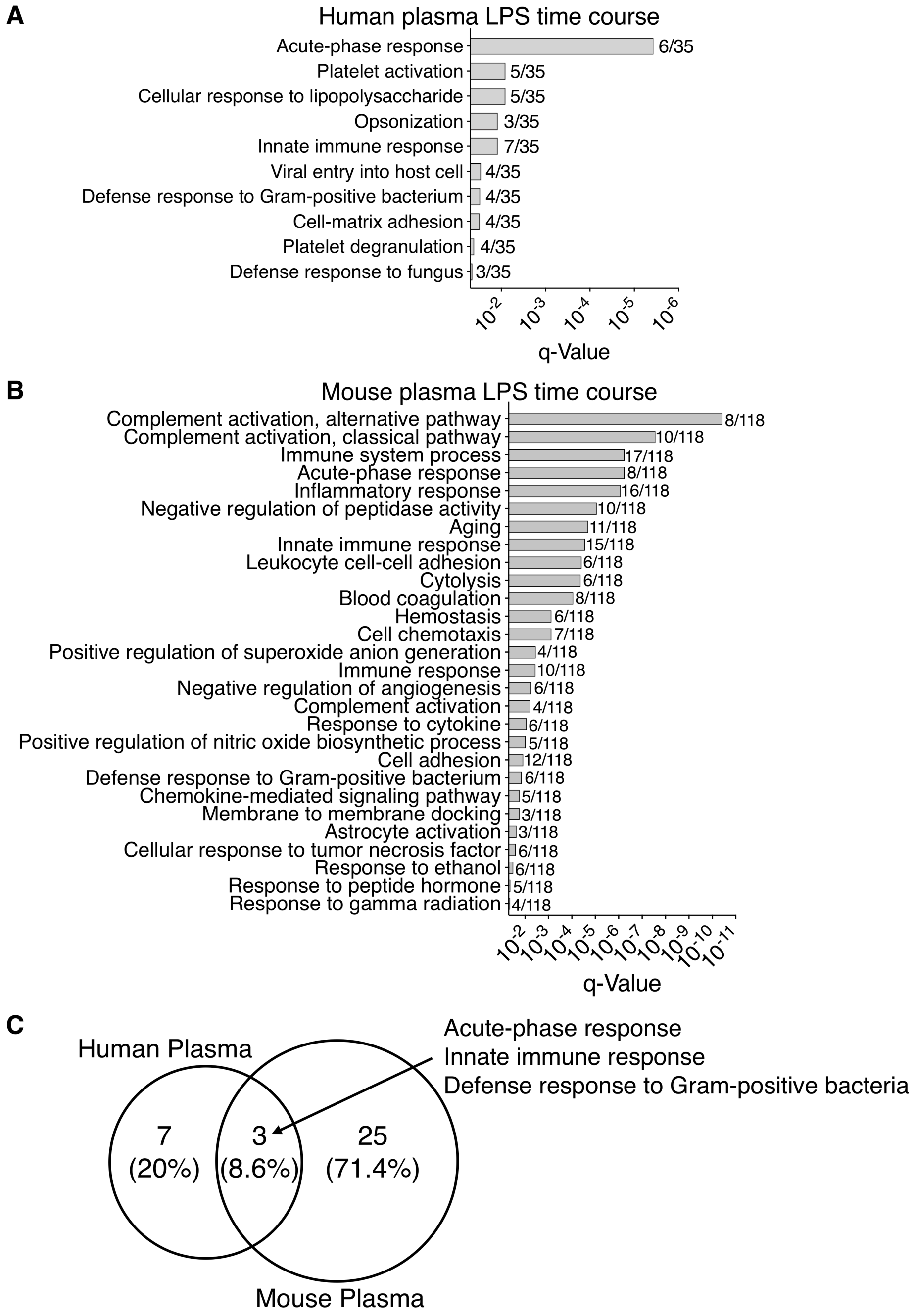

Supplemental Figure 4. ***A comparison between human and mouse plasma proteome responses to LPS***. Gene ontology (GO) enrichment analysis was conducted upon significantly changing proteins ($p<0.05$) in human (**A**) and mouse (**B**) plasma over the LPS time course. Common pathways observed in both humans and mice are displayed in a Venn diagram (**C**).

Table S1. Immunoblot antibody information

Target protein	Host species	Application (dilution)	Company (catalogue number)
Pentraxin-3 (PTX3)	Rabbit	Immunoblotting (1:1000)	Abcam (ab125007)
HRP-anti-Rabbit	Mouse	Immunoblotting (1:5000)	Jackson Immuno Research (211-032-171)

Table S2. TaqMan assays for qPCR

Target gene	Corresponding protein ID	Assay ID
Cd4	CD4_MOUSE	Mm00442754_m1
Cd68	CD68_MOUSE	Mm03047343_m1
Cd8a	CD8A_MOUSE	Mm01182107_g1
Ly6g	LY6G_MOUSE	Mm04934123_m1
Ppbp	CXCL7_MOUSE	Mm00470163_m1
Ptprc	PTPRC_MOUSE	Mm01293577_m1

Table S3. Baseline characteristics of healthy volunteers

	Control arm (N=10)
Age - median years (interquartile range)	22.5 (21.0 - 24.25)
Male sex (%)	100
Weight (kg) - mean (SEM)	76.4 (2.7)
Body mass index (kg/m^2) - mean (SEM)	23.0 (0.6)
Caucasian (%)	10/10 (100)

Table S4. Protein changes in DIA analysis of human plasma during the LPS time course

Gene name	Uniprot accession	Cluster	**0hr** - mean (SEM)	**6hr** - mean (SEM)	**24hr** - mean (SEM)	p-value	q-value
AXL	P30530	Cluster 1	-0.75 (0.12)	1.21 (0.18)	-0.61 (0.12)	4.00E-10	6.57E-08
CD163	Q86VB7	Cluster 1	-0.78 (0.13)	1.03 (0.27)	-0.24 (0.18)	3.89E-06	1.75E-04
CTSB	P07858	Cluster 1	-0.95 (0.11)	0.49 (0.25)	0.46 (0.29)	1.68E-04	5.53E-03
DEFA1	P59665	Cluster 1	-0.68 (0.08)	0.81 (0.38)	-0.13 (0.18)	1.09E-03	2.98E-02
ICAM1	P05362	Cluster 1	-0.99 (0.16)	0.58 (0.23)	0.41 (0.26)	5.78E-05	2.03E-03
IL1RL1	Q01638	Cluster 1	-1.80 (0.12)	0.44 (0.30)	-0.82 (0.27)	3.46E-06	1.71E-04
PTX3	P26022	Cluster 1	-1.62 (0.16)	-0.25 (0.40)	-1.82 (0.19)	6.92E-04	2.13E-02
S100A9	P06702	Cluster 1	-0.64 (0.21)	1.09 (0.24)	-0.45 (0.12)	1.65E-06	1.16E-04
TIMP1	P01033	Cluster 1	-1.00 (0.17)	0.89 (0.25)	0.10 (0.15)	1.51E-06	1.16E-04
VWF	P04275	Cluster 1	-0.65 (0.06)	1.02 (0.35)	-0.36 (0.09)	1.64E-05	6.70E-04
COL3A1	P02461	Cluster 2	-0.35 (0.13)	-0.83 (0.12)	1.03 (0.30)	2.25E-06	1.23E-04
CRP	P02741	Cluster 2	-0.98 (0.25)	-0.5 (0.01)	0.99 (0.35)	1.77E-05	6.70E-04
FCAMR	Q8WWV6	Cluster 2	-1.56 (0.12)	-0.96 (0.21)	0.62 (0.32)	1.32E-06	1.16E-04
LBP	P18428	Cluster 2	-0.69 (0.06)	-0.13 (0.14)	0.82 (0.40)	7.70E-04	2.23E-02
LRG1	P02750	Cluster 2	-1.11 (0.11)	0.15 (0.19)	0.96 (0.16)	3.86E-09	4.75E-07
SAA1	P0DJI8	Cluster 2	-0.82 (0.01)	-0.40 (0.02)	1.23 (0.23)	8.94E-11	2.20E-08
SAA2	P0DJI9	Cluster 2	-0.83 (0.03)	-0.47 (0.04)	1.30 (0.16)	2.32E-14	1.14E-11
SERPINA3	P01011	Cluster 2	-0.64 (0.22)	-0.44 (0.13)	1.09 (0.23)	1.98E-06	1.22E-04

*DIA data was searched in Spectronaut and statistical analysis was conducted in Perseus
*Significance was determined using the in-built ANOVA function with FDR correction
*z-scored protein abundances are provided

Table S5. Protein changes in TMT analysis of mouse plasma during the LPS time course

Gene name	Uniprot accession	Cluster	**Ctrl** - mean (SEM)	**1hr** - mean (SEM)	**2hr** - mean (SEM)	**3hr** - mean (SEM)	**4hr** - mean (SEM)	p-value	q-value
Cxcl2	P10889	Cluster 1	-2.01 (0.22)	0.67 (0.46)	0.63 (0.29)	-0.49 (0.25)	-1.25 (0.15)	2.76E-04	8.33E-03
Col3a1	P08121	Cluster 2	1.61 (0.57)	0.25 (0.2)	-0.14 (0.19)	-0.72 (0.07)	-1.03 (0.1)	2.83E-04	7.69E-03
Sepp1	P70274	Cluster 2	0.8 (0.14)	-1.18 (0.2)	-0.63 (0.32)	0.71 (0.19)	0.89 (0.33)	1.78E-04	8.00E-03
Sele	Q00690	Cluster 2	0.85 (0.08)	-1.37 (0.1)	-0.41 (0.26)	0.52 (0.1)	1.01 (0.3)	7.03E-06	2.41E-03
Dpp4	P28843	Cluster 2	0.32 (0.36)	-1.1 (0.09)	-0.69 (0.11)	0.67 (0.18)	1.39 (0.25)	7.95E-06	2.41E-03
Bche	Q03311	Cluster 2	1.43 (0.4)	-0.95 (0.14)	-0.72 (0.11)	0.13 (0.3)	0.67 (0.34)	1.30E-04	8.00E-03
Ccl2	P10148	Cluster 3	-1.27 (0.06)	-0.82 (0.06)	0.41 (0.27)	0.88 (0.39)	0.93 (0.36)	1.51E-04	6.67E-03
Cd14	P10810	Cluster 3	-0.99 (0.07)	-0.79 (0.06)	-0.09 (0.12)	0.68 (0.56)	1.49 (0.16)	6.78E-05	6.40E-03
Cxcl10	P17515	Cluster 3	-0.89 (0.01)	-0.79 (0.14)	-0.19 (0.14)	0.73 (0.51)	1.46 (0.36)	2.08E-04	8.00E-03
Ftl1	P29391	Cluster 3	-0.59 (0.18)	-0.79 (0.14)	1.52 (0.2)	-0.01 (0.34)	-0.35 (0.41)	1.43E-04	7.00E-03
Icam1	P13597	Cluster 3	-0.75 (0.25)	-0.8 (0.08)	-0.43 (0.09)	1.06 (0.46)	1.33 (0.21)	3.97E-05	6.67E-03
Igfbp1	P47876	Cluster 3	-1.43 (0.02)	-0.69 (0.15)	0.86 (0.24)	0.49 (0.28)	0.72 (0.42)	1.07E-04	7.33E-03
Mpo	P11247	Cluster 3	-1.27 (0.09)	-0.85 (0.13)	0.58 (0.34)	0.54 (0.27)	1.09 (0.17)	6.40E-05	7.00E-03
Spint1	Q9R097	Cluster 3	-1.05 (0.08)	-0.92 (0.29)	0.8 (0.24)	1.05 (0.37)	0.15 (0.3)	3.86E-04	9.71E-03

*TMT data was searched in Proteome Discoverer and statistical analysis was conducted in Perseus
*Significance was determined using the in-built ANOVA function with FDR correction
*z-scored protein abundances are provided

Table S6. Protein changes in TMT analysis of murine aortas during the LPS time course

Gene name	Uniprot accession	Cluster	**Ctrl** - mean (SEM)	**1hr** - mean (SEM)	**2hr** - mean (SEM)	**3hr** - mean (SEM)	**4hr** - mean (SEM)	p-value	q-value
Acadsb	Q9DBL1	Cluster 1	1.49 (0.09)	0.27 (0.38)	-0.11 (0.17)	-0.30 (0.33)	-1.30 (0.31)	3.43E-04	1.00E-01
Dhrs1	Q99L04	Cluster 1	1.31 (0.23)	0.62 (0.36)	-0.57 (0.18)	0.00 (0.42)	-1.17 (0.29)	9.67E-04	1.55E-01
Iars2	Q8BIJ6	Cluster 1	1.86 (0.23)	-0.39 (0.20)	-0.33 (0.20)	-0.28 (0.26)	-0.73 (0.21)	5.06E-05	3.84E-02
Pck2	Q8BH04	Cluster 1	1.67 (0.20)	0.07 (0.13)	-0.59 (0.20)	0.03 (0.41)	-0.99 (0.18)	1.26E-04	6.27E-02
Scpep1	Q920A5	Cluster 1	1.29 (0.26)	0.91 (0.12)	-0.75 (0.31)	-0.40 (0.4)	-0.80 (0.13)	5.11E-04	1.29E-01
Apoa2	P09813	Cluster 2	-1.16 (0.17)	-0.45 (0.32)	0.41 (0.33)	-0.36 (0.17)	1.44 (0.30)	7.15E-04	1.35E-01
Apoc1	P34928	Cluster 2	-1.30 (0.23)	-0.63 (0.38)	0.54 (0.08)	0.00 (0.33)	1.23 (0.40)	6.76E-04	1.38E-01
Apoc3	P33622	Cluster 2	-1.08 (0.14)	-0.55 (0.36)	0.30 (0.23)	-0.29 (0.25)	1.53 (0.38)	6.29E-04	1.43E-01
Gbp2	Q9Z0E6	Cluster 2	-0.49 (0.12)	-0.75 (0.16)	-0.69 (0.10)	0.44 (0.22)	1.72 (0.25)	3.31E-06	5.33E-03
Ifi204	P0DOV2	Cluster 2	-0.72 (0.13)	-0.79 (0.14)	-0.14 (0.31)	-0.02 (0.12)	1.73 (0.36)	2.11E-04	7.83E-02
Ptx3	P48759	Cluster 2	-0.97 (0.04)	-1.01 (0.08)	-0.24 (0.07)	0.99 (0.21)	1.31 (0.22)	2.78E-07	3.46E-04
Saa3	P04918	Cluster 2	-1.01 (0.28)	-0.79 (0.02)	-0.51 (0.04)	0.24 (0.15)	1.72 (0.32)	5.63E-06	9.00E-03
Vcam1	P29533	Cluster 2	-1.17 (0.03)	-0.86 (0.08)	-0.08 (0.12)	0.60 (0.08)	1.54 (0.12)	8.99E-09	2.24E-05

*TMT data was searched in Proteome Discoverer and statistical analysis was conducted in Perseus
*Significance was determined using the in-built ANOVA function with FDR correction
*z-scored protein abundances are provided

Table S7. Protein changes in TMT analysis of murine aortas after neutrophil depletion

Gene name	Uniprot accession	Cluster	**Non-depleted** - mean (SEM)	**Neutrophil depleted** - mean (SEM)	p-value
Cma1	P21844	Cluster 1	-0.74 (0.24)	0.92 (0.22)	1.69E-03
Col4a2	P08122	Cluster 1	-0.61 (0.20)	0.76 (0.49)	2.71E-02
Fbn1	Q61554	Cluster 1	-0.59 (0.21)	0.73 (0.51)	3.53E-02
Gm10393	Q8K262	Cluster 1	-0.62 (0.21)	0.78 (0.46)	2.12E-02
Hp	Q61646	Cluster 1	-0.69 (0.17)	0.87 (0.39)	6.05E-03
Klkb1	P26262	Cluster 1	-0.62 (0.16)	0.78 (0.49)	2.09E-02
Lama2	Q60675	Cluster 1	-0.60 (0.11)	0.75 (0.54)	2.86E-02
Lamb1	P02469	Cluster 1	-0.65 (0.14)	0.81 (0.48)	1.43E-02
Ltbp1	Q8CG19	Cluster 1	-0.60 (0.17)	0.75 (0.52)	3.10E-02
Ltbp2	O08999	Cluster 1	-0.61 (0.11)	0.76 (0.54)	2.59E-02
Mfap2	P55002	Cluster 1	-0.60 (0.18)	0.75 (0.51)	3.13E-02
Nid1	P10493	Cluster 1	-0.63 (0.23)	0.79 (0.43)	1.79E-02
Nid2	O88322	Cluster 1	-0.66 (0.15)	0.83 (0.45)	1.14E-02
Npnt	Q91V88	Cluster 1	-0.62 (0.16)	0.77 (0.51)	2.39E-02
Bcam	Q9R069	Cluster 2	0.69 (0.23)	-0.86 (0.35)	6.43E-03
Cap1	P40124	Cluster 2	0.58 (0.36)	-0.73 (0.34)	3.61E-02
Capg	P24452	Cluster 2	0.58 (0.36)	-0.72 (0.36)	3.97E-02
Hsp90ab1	P11499	Cluster 2	0.61 (0.36)	-0.76 (0.31)	2.64E-02
Myh11	O08638	Cluster 2	0.70 (0.32)	-0.87 (0.17)	5.44E-03
Myh9	Q8VDD5	Cluster 2	0.58 (0.36)	-0.73 (0.34)	3.65E-02
Nucb2	P81117	Cluster 2	0.58 (0.28)	-0.73 (0.46)	3.82E-02
Ptx3	P48759	Cluster 2	0.58 (0.35)	-0.73 (0.35)	3.68E-02
S100a8	P27005	Cluster 2	0.70 (0.33)	-0.88 (0.13)	4.87E-03
S100a9	P31725	Cluster 2	0.70 (0.33)	-0.88 (0.08)	4.47E-03
Sdf2	Q9DCT5	Cluster 2	0.63 (0.38)	-0.79 (0.21)	2.00E-02

*TMT data was searched in Proteome Discoverer and statistical analysis was conducted in Perseus
*Significance was determined using the in-built Student T-test function
*z-scored protein abundances are provided
